# Th22 Cells as Well as Th17 Cells Expand Differentially in Patients with Early-Stage and Late-Stage Myelodysplastic Syndrome

**DOI:** 10.1371/journal.pone.0051339

**Published:** 2012-12-07

**Authors:** Lin-lin Shao, Lei Zhang, Yu Hou, Shuang Yu, Xin-guang Liu, Xiao-yang Huang, Yuan-xin Sun, Tian Tian, Na He, Dao-xin Ma, Jun Peng, Ming Hou

**Affiliations:** 1 Department of Hematology, Qilu Hospital, Shandong University, Jinan, China; 2 Department of Orthopedics, Shandong Provincial Qianfoshan Hospital, Shandong University, Jinan, China; 3 Medical College, Shandong University, Jinan, China; 4 Department of Pediatrics, Qilu Hospital, Shandong University, Jinan, China; Cincinnati Children’s Hospital Medical Center, United States of America

## Abstract

**Background:**

Immunological mechanisms are increasingly recognized in the progression of myelodysplastic syndrome (MDS). Early-stage MDS (E-MDS) is characterized by autoimmune-mediated myelosuppression whereas late-stage MDS (L-MDS) involves immune evasion, giving dysplastic cells growth potential to progress into acute myeloid leukemia. T-helper (Th) 22 is involved in the pathogenesis of inflammatory autoimmunity and tumorigenesis. The roles of Th22 cells in the pathophysiology of E-MDS and L-MDS remain unsettled.

**Design and Methods:**

We studied 37 MDS patients (E-MDS, n = 17; L-MDS, n = 20) and 20 healthy controls to characterize their peripheral blood (PB), as well as 25 MDS patients and 10 healthy controls to characterize their bone marrow(BM). The expression of Interleukin-22 (IL-22), IL-17 or interferon gamma (IFN-γ) was examined in E-MDS, L-MDS patients and controls by flow cytometry. The mRNA expression levels of RAR-related orphan receptor C (*RORC*), *IL-6, tumor necrosis factor alpha (TNF-α)* and *IL-23* in peripheral blood mononuclear cells (PBMCs) were determined by real-time quantitative polymerase chain reaction. The levels of IL-22 and IL-17 both in PB and BM plasma were examined by enzyme-linked immunosorbent assay.

**Results:**

In E-MDS, peripheral Th17 cells were significantly elevated and correlated with peripheral Th22 cells compared with healthy controls and L-MDS. Significantly higher levels of peripheral Th22 expansion, mRNA expression of *IL-6, TNF-α* and lower level of *RORC* mRNA expression were observed in L-MDS compared with E-MDS. No statistical difference was found in *IL-23* mRNA expression or plasma IL-22, IL-17 levels among E-MDS, L-MDS and controls.

**Conclusions:**

Our data demonstrated that L-MDS cohort had increased frequencies of peripheral Th22 cells and higher mRNA expression levels of *IL-6* and *TNF-α*, indicating that Th22 cells along with Th17 cells or not are involved in the dynamic immune responses of MDS.

## Introduction

Primary myelodysplastic syndrome (MDS) encompasses a heterogeneous group of clonal hematopoietic stem-cell disorders, characterized by ineffective hematopoiesis and an increased probability of developing acute leukemia. Autoimmune-mediated myelosuppression and immune evasion of malignant clone are increasingly recognized in the process of MDS. Clinical responses to immunoregulatory therapy and findings of expanded clonal T lymphocytes have led to the speculation that both CD4^+^ helper and CD8^+^ cytotoxic T cells are involved in the immunological pathophysiology of MDS [1], [2], [3].

Recently, a separate CD4^+^ T-cell subset was identified on the basis of cytokine interleukin(IL)-17 [4]. This subset was subsequently named T-helper cell type17 (Th17), expressing the lineage-specific transcription factor retinoic acid receptor-related orphan receptor C (*RORC*) [5]. Since then, Th17 cells have entered the limelight because of their comprehensive involvement in inflammations [6]. IL-17A, a representative Th17 cytokine, has been described in various models of immune-mediated tissue injury, including rheumatoid arthritis, lupus, myeloma as well as others likely to be defined. Though Kordasti and colleagues recently showed the increased number of peripheral Th17 cells in low risk MDS [7],the mechanism of cellular immune abnormalities remains unclear.

Th22, capable of secreting IL-22 and tumor necrosis factor alpha (TNF-α), is a new subset of T cells clearly separated from other known Th cells [8]. This subset expresses none of interferon (IFN)-γ, IL-17 cytokine, or respectively associated transcription factors T-bet and RORC [9]. Promoted by IL-6 and TNF-α, aryl hydrocarbon receptor (AHR) activation participates in priming human naive CD4^+^ T cells to Th22 subset [8], [10]. Th22 cells have been found within the epidermal layer in prominence and regulating epidermal responses in inflammatory skin diseases [8]. In addition, Zhuang et al. have described a preferential expansion of Th22 cells which contribute to gastric cancer progression [11]. These data support the contention that Th22 cells are involved in the pathophysiology of autoimmunity and tumorigenesis.

IL-22 belongs to the IL-10 family of cytokines and is primarily secreted by activated Th22 cells [12]. The expression of IL-22 in cancers and autoimmune disorders is various, with IL-17 as siblings but not twins regarding their biological characteristics. IL-22 was up-regulated in skin pathology and anaplastic lymphoma kinase positive anaplastic large cell lymphoma, providing signaling directionality from the immune system to targeted tissue-resident cells [12], [13], [14]. Meanwhile, it was down-regulated in systemic lupus erythematosus [15]. However, within disorders such as inflammatory bowel disease (IBD), IL-22 played either protective or pathogenic role in discrepant induction by naive and memory/effector cells [16], [17].

Up to now, no data exist with regard to Th22 cells and their association with Th17 or Th1 in MDS patients. To investigate possible roles of the above in the pathophysiology of MDS, we measured the percentages of peripheral Th22, Th17, Th1, mRNA expression levels of *RORC*, *IL-6, TNF-α* and *IL-23* in peripheral blood mononuclear cells (PBMCs) as well as cytokine level of IL-22 or IL-17 in peripheral blood (PB) and bone marrow (BM), and evaluated their relevance.

## Materials and Methods

### Ethics Statement

Our research has been approved by the Institutional Review Boards of Qilu Hospital of Shandong University. A written informed consent document has been obtained from each participant. The informed consent stated that the excess of peripheral blood for Flow Cytometry - Clinical Diagnostics or unused portion of bone marrow for Fluorescence in situ hybridization (FISH) - Clinical Diagnostics was the sample source of our research. The peripheral blood drawn from healthy subjects and bone marrow drawn from hematologically normal individuals undergoing orthopedic femoral surgery for this research was voluntary.

### Patients and Controls

A total of 54 patients (15 females and 39 males; mean age 52.6±15.4 years) with MDS according to the World Health Organization (WHO) classification [18] were recruited in this study. All patients were treatment-naive or had no medical interventions for at least 3 months when sampling. Twenty age-matched healthy PB donors (6 females and 14 males; mean age 51.0±15.9 years) were also included in the study. Ten hematologically normal age-matched individuals (3 females, 7 males; mean age 53.1±11.8 years) were used as BM controls. Enrollment took place between March 2011 and May 2012 in the Department of Hematology of Qilu Hospital, Shandong University, China. FISH was performed to record cytogenetic karyotype regarding 5q31, 5q33, CEP7, 7q31, CEP8, 20q and CEPY. The patients were further divided into two subgroups based on International Prognostic Scoring System (IPSS) score [19], early-stage MDS (E-MDS, low/intermediate-1 risk, n = 29) and late-stage MDS (L-MDS, intermediate-2/high risk, n = 25). 5% BM blasts was chosen as the cut-off value delimiting fifty-six percent of MDS patients <5% and forty-four percen t ≥5%. The demographic and key clinical features of MDS patients are listed in Table 1.

**Table 1:** Demographic and clinical characteristics of MDS patients.

Characteristics	value
No. of patients	54
Age(y)	52.6±15.4
Sex(male/female)	39/15
IPSS risk group, n(%)	
E-MDS: Low+Intermediate-1	29 (53.7%)
L-MDS: Intermediate-2+ High	25 (46.3%)
WHO MDS category, n(%)	
Unknown	15(27.7%)
RCUD	13(24.1%)
RARS	1(0.2%)
RCMD	2(3.7%)
RAEB-1	6(11.1%)
RAEB-2	13(24.1%)
MDS/MPD	2(3.7%)
CMML-1	1(0.2%)
CMML-2	1(0.2%)
BM blasts, n(%)	
<5	30(55.6%)
≥5	24(44.4%)
IPSS karyotype, n(%)	
Favorable	33(61.1%)
Intermediate	12(22.2%)
Unfavorable	9(16.7%)

Abbreviations: BM, bone marrow; n, number; RCUD, refractory cytopenia with unilineage dysplasia; RARS, refractory anemia with ring sideroblasts; RCMD, refractory cytopenia with multilineage dysplasia; RAEB, refractory anemia with excess blasts; CMML, chronic myelo-monocytic leukemia.

### Preparation of Peripheral Blood Mononuclear Cells, Blood and Bone Marrow Plasma

Peripheral whole blood was collected from 37 patients (E-MDS, n = 17; L-MDS, n = 20) while bone marrow were drawn from 25 cases. Plasma was obtained by centrifugation of heparinized peripheral blood and stored at −80°C for cytokine analysis. Peripheral blood mononuclear cells (PBMCs) were isolated from EDTA anticoagulated blood by gradient centrifugation (400 g, 20 minutes) using Ficoll-Paque (Pharmacia Diagnostics) and stored at −80°C for RNA isolation.

### Flow Cytometric Analysis

Intracellular cytokines were studied by flow cytometry to reflex the cytokine-producing cells. Briefly, heparinized peripheral whole blood (400 µl) with an equal volume of Roswell Park Memorial Institute 1640 medium was incubated for 4h at 37°C, 5% CO_2_ in the presence of 25 ng/ml of phorbol myristate acetate (PMA), 1 µg/ml of ionomycin, and 1.7 µg/ml Golgiplug (monensin; all from Alexis Biochemicals, San Diego, CA, USA). PMA and ionomycin are pharmacological T-cell-activating agents that mimic signals generated by the T-cell receptor (TCR) complex and have the advantage of stimulating T cells of any antigen specificity. Monensin was used to block the intracellular transport mechanisms, thereby leading to an accumulation of cytokines in the cells. After incubation, cells were stained with PE-Cy5-conjugated anti-CD4 monoclonal antibody at room temperature in the dark for 20 min. The cells were next stained with FITC-conjugated anti-IFN-γ monoclonal antibody, PE-conjugated anti-IL-17 monoclonal antibody, and APC-conjugated anti-IL-22 monoclonal antibody after fixation and permeabilization. All antibodies above were from eBioscience (SanDiego, CA, USA). Isotype controls were given to enable correct compensation and confirm antibody specificity. Stained cells were analyzed by flow cytometric analysis using a FACS Calibur cytometer equipped with CellQuest software (BD Bioscience PharMingen).

### Determination of the Expression of *RORC*, *IL-6*, *TNF-α*, *IL-23* mRNA

TRIzol reagent (Invitrogen) was used to isolate total RNA of PBMCs. RNA was converted into cDNA using the PrimeScript RT reagent kit (Perfect Real Time; Takara) according to the manufacturer’s instructions. Real-time polymerase chain reaction (PCR) was performed for *RORC*, *IL-6*, *TNF-α*, *IL-23* and the endogenous control (*β-actin*) on an ABI 7500 Real-time PCR System (Applied Biosystems) using SYBR Green (Toyobo) as a double-strand DNA-specific binding dye. The primers for all mRNA arrays were intron spanned. The PCR reactions were cycled 40 times after initial denaturation (95°C, 5 minutes) with the following parameters: denaturation at 95°C for 15 seconds, annealing at 65°C (*RORC, β-actin*)/62°C(*IL-6, TNF-α*, *IL-23, β-actin*)for 15 seconds, extension at 72°C for 45 seconds. The primers are shown as follows: *RORC* forward: TTT TCC GAG GAT GAG ATT GC; reverse: CTT TCC ACA TGC TGG CTA CA; *IL-6* forward: TTC TCC ACA AGC GCC TTC GGT CCA, reverse: AGG GCT GAG ATG CCG TCG AGG ATG TA; *TNF-α* forward: CGA GTG ACA AGC CTG TAG C, reverse: GGT GTG GGT GAG GAG CAC AT; *IL-23* forward: GCA GAT TCC AAG CCT CAG TC, reverse: TTC AAC ATA TGC AGG TCC CA; *β-actin* forward: CCT TCC TGG GCA TGG AGT CCT G, reverse: GGA GCA ATG ATC TTG ATC TTC. The amplification efficiency of the PCR products was calculated using Applied Biosystems System software. Target gene concentrations were expressed relative to the number of β-actin transcripts used as the reference control. All experiments were conducted in triplicate.

### IL-22 and IL-17 Enzyme-Linked Immunosorbent Assay (ELISA)

PB and BM were collected into heparin-anticoagulant vacutainer tubes. Plasma was obtained by centrifugation and stored at −80°C for determination of cytokines. IL-22 and IL-17 levels were determined with a quantitative sandwich enzyme immunoassay technique in accordance with the manufacturer's recommendations (lower detection limit 9 pg/ml; eBioscience).

### Statistical Analysis

Results were expressed as mean ± SD or median (range). Comparisons between two groups were assessed by the non-paired t-test or the Wilcoxon rank-sum test to compare parametric and non-parametric data respectively. Statistical significance among three groups was determined by ANOVA, and difference between any two groups was determined by Newman–Keuls multiple comparison test (q test) unless the data were not normally distributed, in which case Kruskal–Wallis test (H test) and Nemenyi test were used. The Pearson or Spearman correlation test was used for correlation analysis depending on data distribution. All tests were performed by SAS 9.1 system. P value less than 0.05 was considered statistically significant.

## Results

### Elevated Circulating Th22 Cells in MDS Patients

**Figure 1:**
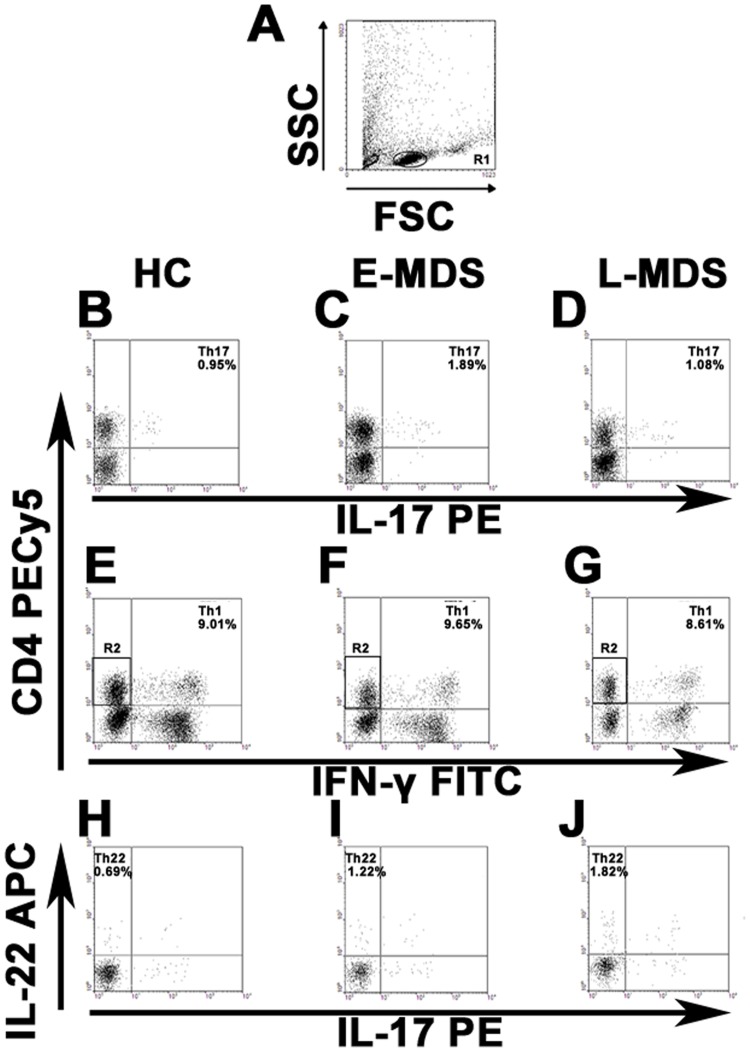
Circulating percentages of Th17, Th1 and Th22 cells in representative healthy controls, E-MDS and L-MDS patients. Heparinized peripheral whole blood from 37 MDS(E-MDS, n = 17; L-MDS, n = 20)patients and 20 healthy PB donors were stimulated with phorbol myristate acetate, ionomycin, and monensin for 4 h, and then stained with labeled antibodies for FACS analysis. (A) Lymphocytes were gated by flow cytometry. (B, C, D) Representative FACS dot plots of circulating Th17 (CD4^+^IL-17^+^) cells from healthy controls, E-MDS and L-MDS patients. (E, F, G) Representative FACS dot plots of circulating Th1 (CD4^+^IFNγ^+^) cells from healthy controls, E-MDS and L-MDS patients. (H, I, J) Representative FACS dot plots of circulating Th22 (CD4^+^IL-22^+^IL-17^−^IFNγ^−^ ) cells from healthy controls, E-MDS and L-MDS patients. Numbers in plots indicate relative percentages per quadrant.

**Figure 2:**
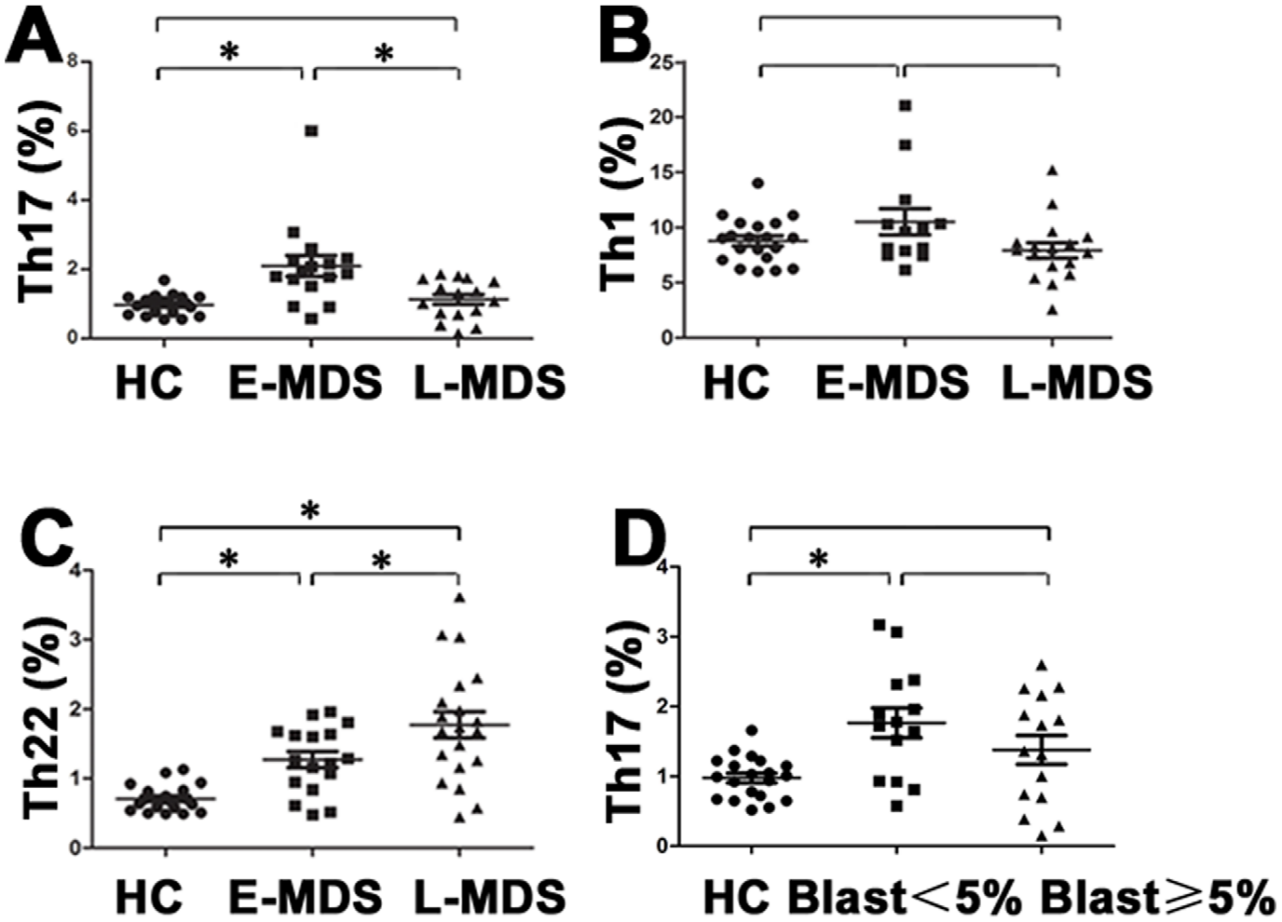
Circulating percentages of Th17 cells, Th1 cells, and Th22 cells in MDS. (A) Circulating percentages of Th17 (CD4^+^ IL-17^+^) cells from healthy controls, E-MDS and L-MDS. Significantly increased percentage of Th17 cells was found in E-MDS patients (median, 1.90%; range, 0.58–6.01%) compared to L-MDS (median, 1.16%; range, 0.15–1.86%) (*P = 0.002) or healthy controls (median, 1.01%; range, 0.55–1.69%) (*P = 0.002). (B) Circulating percentages of Th1 (CD4^+^IFNγ^+^) cells from healthy controls, E-MDS and L-MDS. There was no significant difference between E-MDS (median, 9.65%; range, 6.16–21.08%) patients and L-MDS (median, 8.41%; range, 2.59–15.23%) or healthy controls (median, 9.06%; range, 6.01–14.02%). (C) Circulating percentages of Th22 (CD4^+^ IL-22^+^ IL-17^−^IFNγ^−^ ) cells from healthy controls, E-MDS and L-MDS. Significantly elevated percentage of Th22 cells was found in L-MDS patients (1.77±0.84%) compared to E-MDS (1.27±0.50%) (*P = 0.03) and healthy controls (0.71±0.17%) (*P<0.0001). Obviously increased percentage of Th22 cells was shown between E-MDS and healthy controls (*P = 0.002). (D) Circulating percentages of Th17 cells from healthy controls, bone marrow (BM) blasts <5% and blasts ≥5% patients. The circulating percentages of Th17 cells remained significantly higher in blasts <5% compared with healthy donors. (*P = 0.003).

**Figure 3:**
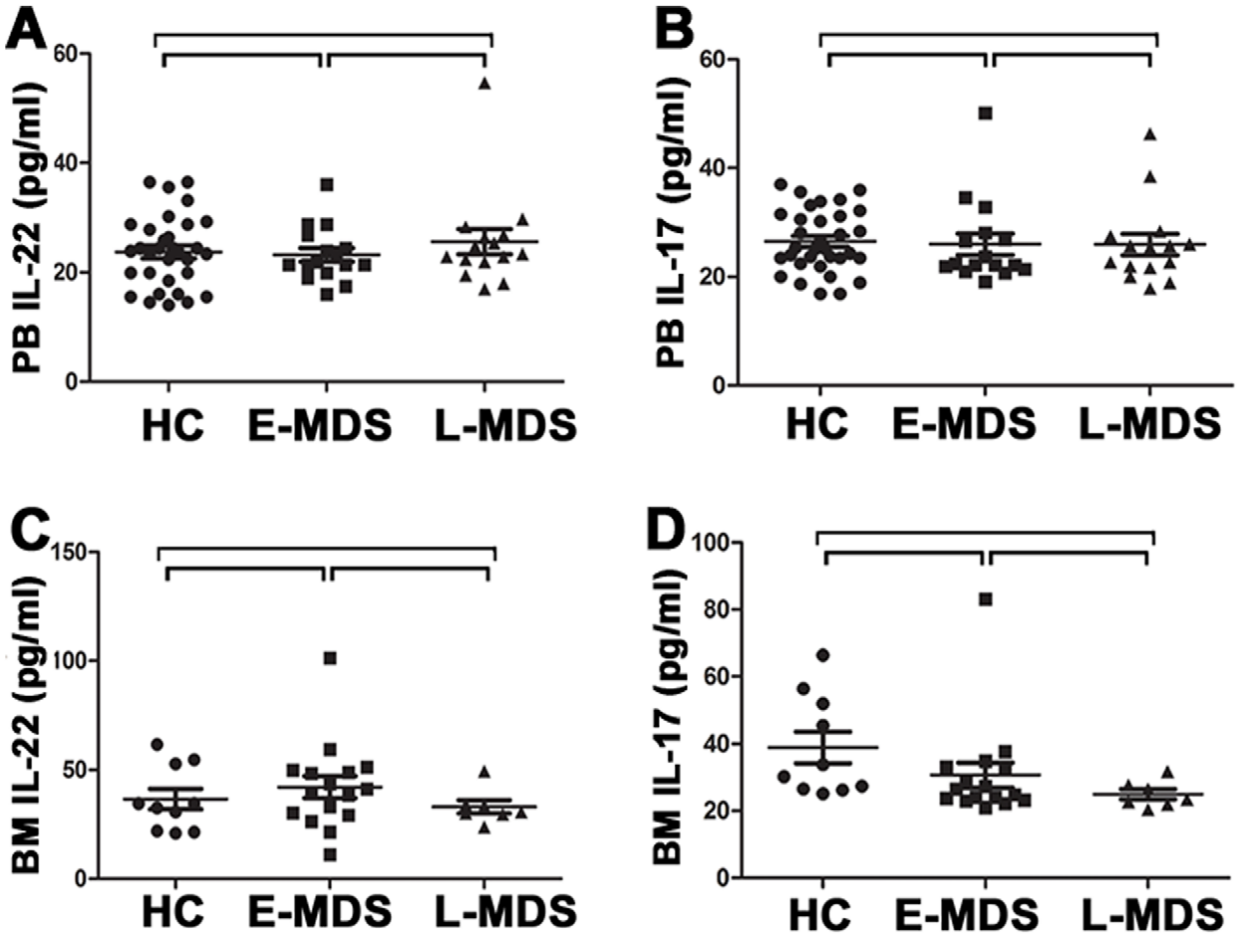
Concentrations of IL-22 and IL-17 in PB and BM from healthy controls and MDS patients. (A) Concentrations of IL-22 in PB plasma from healthy controls, E-MDS and L-MDS patients. There was no significant difference between E-MDS (median, 21.66 pg/ml; range, 16.02–36.00 pg/ml, *P*>0.05) or L-MDS patients (median, 23.37 pg/ml; range, 17.00–54.66 pg/ml, *P*>0.05) and healthy controls (median, 23.86 pg/ml; range, 14.04–36.49 pg/ml, *P*>0.05). (B) Concentrations of IL-17 in PB plasma from healthy controls, E-MDS and L-MDS patients. No significant difference was found between E-MDS (median, 22.32 pg/ml; range, 19.13–50.11 pg/ml, *P*>0.05) or L-MDS (median, 25.39 pg/ml; range, 17.86–46.31 pg/ml, *P*>0.05) patients and healthy controls (median, 25.11 pg/ml; range, 16.87–37.00 pg/ml, *P*>0.05).(C)Concentrations of IL-22 in BM plasm from healthy controls, E-MDS and L-MDS patients. There was no significant difference between E-MDS (median, 40.34 pg/ml; range, 11.08–101.33 pg/ml, *P*>0.05) or L-MDS patients (median, 30.68 pg/ml; range, 23.86–49.43 pg/ml, *P*>0.05) and healthy controls (median, 33.59 pg/ml; range, 20.93–61.74 pg/ml, *P*>0.05). (D) Concentrations of IL-17 in BM plasm from healthy controls, E-MDS and L-MDS patients. No significant difference was found between E-MDS (median, 25.68 pg/ml; range, 20.96–83.13 pg/ml, *P*>0.05) or L-MDS patients (median, 23.48 pg/ml; range, 20.49–31.80 pg/ml, *P*>0.05) and healthy controls (median, 31.99 pg/ml; range, 25.11–66.37 pg/ml, *P*>0.05).

We analyzed the frequency of peripheral Th22 based on cytokine patterns after in vitro stimulation by PMA plus ionomycin in short-term cultures. The expression of a typical dot plot of circulating Th22 (CD4^+^IL-22^+^IL-17^−^IFNγ^−^) cells in representative MDS patients and healthy controls (HC) is shown in Fig. 1 H, I, J. Compared with HC, the percentage of peripheral Th22 cells was significantly increased in MDS patients (0.71±0.17% vs. 1.55±0.74%, *P*<0.0001). The percentage of peripheral Th22 cells in E-MDS is higher than that in controls(1.27±0.50% vs. 0.71±0.17%, *P* = 0.002). Also a significant increase was shown in L-MDS compared with E-MDS patients (1.77±0.84% vs. 1.27±0.50%, *P* = 0.03) (Fig. 2C). The levels of IL-22 in PB and BM were measured by ELISA. No significant difference of PB IL-22 level between MDS patients (median, 22.64 pg/ml; range, 16.02–54.66) and normal controls (median, 23.86 pg/ml; range, 14.05–36.49) was observed, consistent with BM findings (Fig. 3 A, C). No correlation was found between peripheral Th22 cells and circulating IL-22 in MDS patients. Meanwhile, peripheral Th1 and Th17 cells failed to show a statistical correlation with circulating level of IL-22 in our present research.

### Elevated Circulating Th17 Cells in E-MDS Patients

The frequency of Th17 cells (CD4^+^IL-17^+^) was profoundly increased in MDS patients compared to healthy controls (1.64±1.04% vs. 0.97±0.29%, *P* = 0.002). Further on, a significant increase of peripheral Th17 cells was seen in E-MDS(median, 1.90%; range, 0.58–6.01%)compared with L-MDS(median, 1.16%; range, 0.15–1.86%)(*P* = 0.002) (Fig. 1 B, C, D and Fig. 2A). When compared to HC, E-MDS patients but not L-MDS ones, had a distinctly greater median percentage of committed Th17 cells among peripheral CD4+ cells (*P* = 0.002 vs. *P* = 0.34) (Fig. 2A). However, there was no marked difference regarding peripheral IL-17 between MDS patients (median, 25.1 pg/ml; range, 16.9–50.1) and HC (median, 22.4 pg/ml; range, 17.9–37.0; *P* = 0.74) (Fig. 3B). No significant difference of IL-17 level was shown between BM and matched PB (*P*>0.05) (Fig. 3D). No correlation was identified between peripheral Th17 frequency and circulating IL-17 level.

For peripheral Th1 cells, no significant difference was observed between MDS patients and HC (median, 9.65%; range, 6.16–21.08% vs. median, 9.06%; range, 6.01–14.02%) in this study (Fig. 1 E, F, G and Fig. 2B).

### mRNA Expression Levels of *RORC*, *IL-6*, *TNF-α* and *IL-23* in E-MDS, L-MDS Cohort and Controls

**Figure 4:**
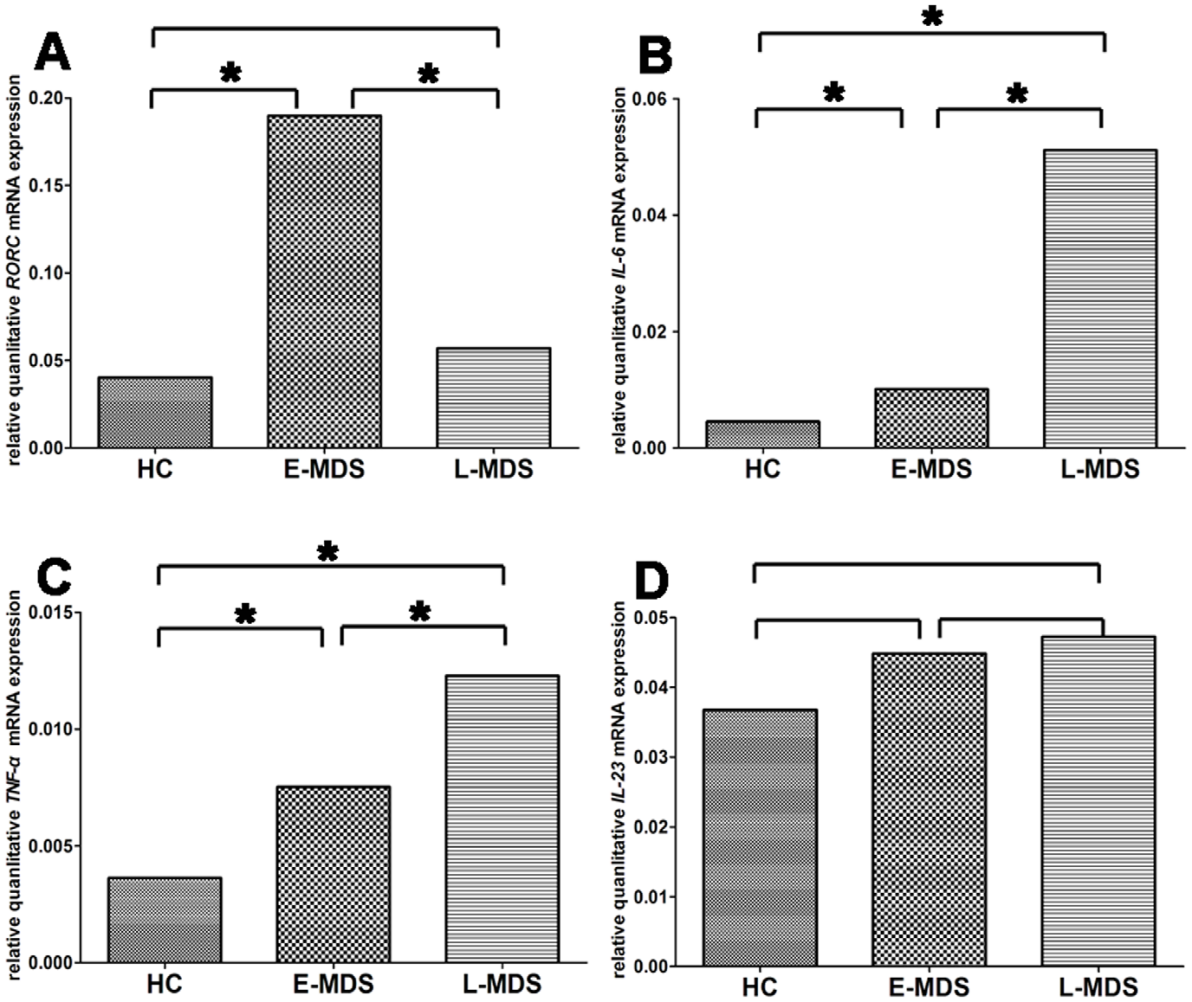
The ratio of *RORC, IL-6, TNF-α, IL-23* mRNA in healthy controls and MDS patients. (A) The ratio of *RORC* mRNA in E-MDS patients compared with that of healthy controls or L-MDS was 4.7 (*P = 0.0007) or 3.3 (*P = 0.002), respectively. (B) The ratio of *IL-6* mRNA in L-MDS patients compared with that of healthy controls or E-MDS was 5.3 (*P = 0.0001) or 2.4 (*P = 0.037), respectively. (C) The ratio of *TNF-α* mRNA in L-MDS patients compared with that of healthy controls or E-MDS was 10.6 (*P = 0.002) or 3.5 (*P = 0.049), respectively. (D) *IL-23p19* mRNA expression level among E-MDS, L-MDS and healthy controls was comparable (P>0.05). Bars represent SD.

*RORC,* the key transcription factor directing Th17 lineage commitment, was determined by real-time PCR. Our result demonstrated that RORC transcript was notably higher in PBMCs of E-MDS patients, consistent with the flow cytometry data. The relative amount of RORC mRNA in E-MDS patients was increased 4.7- and 3.3-fold compared with healthy controls (HC) and L-MDS patients (P = 0.0007; P = 0.002, respectively) (Fig. 4A).

In view of increased frequencies of Th22 cells in L-MDS and Th17 cells in E-MDS, we also determined the mRNA expression levels of regulatory factors *IL-6*, *TNF-α* and *IL-23* in MDS patients and controls. The relative amount of mRNA of *IL-6* in L-MDS patients was 2.4-(P<0.05) and 5.3-fold (P<0.001) of that in E-MDS patients and healthy controls, respectively (Fig. 4B). TNF-α mRNA level was also present at higher levels in L-MDS compared with E-MDS and controls (3.5-fold, P<0.05 and 10.6-fold, P<0.005, respectively) (Fig. 4C).

IL-23 is a heterodimeric cytokine consisting of two subunits: p40, which is also a component of IL-12, and p19, a unique subunit of IL-23. So we examined *IL-23p19* mRNA expression as representative of IL-23. Quantitative real-time PCR analysis showed that there was no significant difference in *IL-23p19* mRNA expression levels among E-MDS, L-MDS and healthy controls (P>0.05) (Fig. 4D).

### Correlation between Th22, Th17, and Th1 Cells in MDS PATIENTS

In E-MDS patients, a significantly positive correlation was found between peripheral Th22 cells and Th17 cells (r = 0.675, *P* = 0.004, Pearson correlation analysis) while no statistical correlation was obtained in L-MDS patients (r = 0.138, *P* = 0.610, Spearman correlation analysis). Peripheral Th22 cells showed no significant correlation with peripheral Th1 (*P* = 0.053).

### Clinical Relevance of Peripheral Th22, Th17 and Circulating IL-22 in MDS Patients

Peripheral Th17 percentage significantly increased in BM blasts <5% patients compared with healthy donors (1.76±0.80% vs. 0.98±0.30%, *P* = 0.003), while no significant difference was seen between BM blasts ≥5% patients and healthy donors (1.38±0.80% vs. 0.98±0.30%, *P*>0.05) or between blasts ≥5% and <5% patients (1.38±0.80% vs. 1.76±0.80%, *P*>0.05) (Fig. 2D). As for the correlations between peripheral Th22 frequency and the levels of three kinds of blood cells in MDS patients, no significant differences were found.

## Discussion

The presence of immune reactions against hematopoietic cells in MDS patients has crystallized as specific cell subsets and cytokines turmoil have been found as indispensable components of MDS pathophysiology. Th17, a new CD4^+^ T cell lineage that preferentially produces IL-17, has been described to be involved in various autoimmune diseases. Currently, there are two reports addressing discrepant ideas that Th17 cells operate to regress or enhance leukemic progression of MDS [7], [20]. Compared with the well-known Th1, Th2 and Th17 subset,another distinct CD4^+^ T cell lineage, capable of secreting IL-22 but not IL-17 or IFN-γ, has been denoted as Th22 subset [9], [12]. Although previous studies have indicated that IL-22 secreted by Th22 participates in certain tumorigenicity and autoimmunity [14], [21], it is not clear whether they are involved in MDS yet.

To study whether Th22 subset is compromised in the process of MDS, the percentages of peripheral Th22 cells in MDS patients and healthy controls were determined. Our results demonstrated that the percentage of peripheral Th22 subset (defined as CD4^+^IL-22^+^IL-17^−^IFNγ^−^) was markedly elevated in MDS patients compared with healthy donors, and notably higher in L-MDS than in E-MDS. These results indicated that Th22 might be more involved in the immune evasion of MDS contributing to disease progression. Facts provided by advanced studies of Th22 cells in infection, inflammation, autoimmunity and cancer suggest that Th22 may play a biphasic role varying on the focal microenvironment [8], [11]. With respect to disordered immune function in preleukemic states, E-MDS and L-MDS can be considered as two separate entities. The former is characterized by excessive apoptotic activity with autoimmune assault in the bone marrow whereas the latter involves decreased apoptotic indices and dramatic suppression of host anti-tumor responses, giving dysplastic cells the growth potential to progress into acute myeloid leukemia [22], [23]. In our present study, increased Th17 cells have been advocated in E-MDS in a pattern reminiscent of autoimmunity, backed up by an analogous result from Mufti’s group [7]. Different from previous report [20], we found elevated *RORC* mRNA expression level in peripheral blood of E-MDS patients compared with normal controls and L-MDS patients, suggesting that the differentiation of Th17 cells takes part in E-MDS pathophysiology specifically. In our present study, no significant difference of IL-17 concentration whether in the BM or PB among E-MDS patients, L-MDS patients, or healthy controls was found. Although IL-23 signaling is dispensable for Th17 commitment, it induces IL-17 production as one of the essential cofactors [24]. Our present study regarding IL-23 mRNA expression level did not show the difference between E-MDS, L-MDS and controls. Along with previous studies regarding the serum level of IL-23 [7], we speculate that the unaltered IL-17 level vs. elevated Th17 cells in E-MDS attributes to IL-23 insufficiency.

In our study, there existed a positive correlation between peripheral Th22 and Th17 subset in E-MDS patients, implying that differentiation of Th22 and Th17 cells may be induced in an influential manner in E-MDS. Co-existence of Th22 and Th17 cells along with pro-inflammatory cytokines leads to a dramatic increase in the overexuberant inflammatory immune reaction [8]. Such functional synergism may happen in the persistent low-level inflammatory state of E-MDS in which elevated levels of pro-apoptotic cytokines, low regulatory T-cells (Tregs) number and increased natural killer (NK) cytotoxicity against hematopoiesis are widely recognized features [25], [26], [27]. On the other hand, L-MDS, a disease stage progressing towards AML with additional genetic lesions, is characterized by increased numbers of Tregs [28], dysfunctional NK cells [29] and higher immunoinhibitory molecule B7-H1 expression on MDS blasts [22], resulting in immune evasion of the malignant clone. Stimulation of IL-6 plus TNF-α could promote Th22-cell differentiation from CD4^+^ T cells [30]. Both TNF-α and IL-6 have been detected excessively expressed in the sera of patients with high risk MDS [31]. Our data demonstrated that L-MDS patients had increased *IL-6* and *TNF-α* mRNA expression compared with E-MDS patients or normal controls. Thus, it can be concluded that a cell-cytokine milieu within the tumor microenvironment of L-MDS may be suitable for the polarization of Th22 cells. IL-22R1, the critical member of IL-22 receptor complex, was not detected in normal immune cells [32]. Notwithstanding, recent study found aberrant expression of IL-22R1 on anaplastic lymphoma kinase positive anaplastic large cell lymphoma (ALK^+^ ALCL) cell lines [14]. Several reports have identified that IL-22 secreted by Th22 cells interacts with CD4^+^ T cells, monocyte-derived human macrophages and bone marrow-derived dendritic cells (DCs) [33], [34], [35]. To some instances, we hypothesize that Th22 cells participate in the immune regulation of hematopoiesis probably through interaction with DCs or other Th subsets. Despite these appearant correlations, at this period we cannot confirm the direct effect of Th22 on the impaired immune surveillance of L-MDS.

Numerous evidences suggest Th1 cells have been linked to the development of autoimmune inflammatory processes. To further investigate the role of Th1 cells in the pathogenesis of MDS, we also examined the percentages of periphery Th1 cells in MDS patients and healthy donors. In contrast to the results of periphery Th22 and Th17 cells, no statistical difference was shown in Th1 frequencies between patients with MDS and healthy donors. Although previous studies showed that the conventional Th1-prototypical cytokines IFN-γ and TNF-α were predominant in MDS, it was later confirmed that these cytokines were derived from macrophage lineage cells [36]. Meanwhile, statistical correlations between Th1 cells and Th22 cells or Th17 cells were not found in this study.

IL-22 has been involved in the pathophysiology of some malignant diseases, such as multiple myeloma [37]. As to MDS, the expression level of IL-22 has not been investigated until today. Our results demonstrated for the first time that the level of IL-22 either in BM or in PB of MDS patients was comparable with that of healthy controls, and no correlation with peripheral Th22 cells was revealed. IL-22 is not exclusively produced by Th22 cells, but rather appears to be produced by other T cells such as CD4^+^IL-17^+^IL-22^+^ cells and CD4^+^IFNγ^+^IL-22^+^ cells, as well as circulating NK cells [38], [39]. The frequency of Th22 cells among total IL-22-producing T cells ranges from 37% to 63% [9]. When taking IL-22-producing NK cells into account, that proportion will become smaller. In MDS, groups have reported that function of MDS-NK cells are reduced and cytokine secretion are decreased [40]. The data above unambiguously indicate that the unilateral increased frequency of Th22 cells contributes little to the IL-22 production capacity. On the other hand, mounting evidences support that IL-6 possesses a developmental relationship to the IL-22 production [24]. Inversely, TGF-β down-regulates the IL-22 production [41]. Compelling evidence has been achieved from several groups that levels of IL-6 as well as TGF-β are increased in MDS patients [42], [43]. So the unaltered extracellular level of IL-22 can be partially attributed to the regulating homeostasis between IL-6 stimulative and TGF-β inhibitory effect.

In summary, for the first time we showed a clear difference between E- and L-MDS in terms of Th22-cell related immunological environment. Circulating Th22 expansion occurs much more frequently in late stage, which may favor the escape of the preleukemic clone. By contrast, in early stage, circulating Th17 expansion tends to be predominant, thereby underpinning the inflammatory autoimmune assault and eventually the apoptosis of bone marrow. The numerical alterations of Th22 subset in early and late disease stage would suggest that shifty in the dynamics of Th22 could be a parameter affecting disease progression, exerting antithetical effects in the regulation of immune homeostasis and tumor immunity. Blockade of Th22 cells might be of clinical profit in both E-MDS and L-MDS patients. Further studies on more patients are needed to substantiate whether this is indeed the case, and it is necessary to clarify the situation of Th22 cells in MDS bone marrow.
